# The contributions of public health policies and healthcare quality to gender gap and country differences in life expectancy in the UK

**DOI:** 10.1186/s12963-021-00271-2

**Published:** 2021-10-20

**Authors:** Kasim Allel, Franceso Salustri, Hassan Haghparast-Bidgoli, Ali Kiadaliri

**Affiliations:** 1 Institute for Global Health, University College London, 30 Guilford Street, London, WC1N 1EH UK; 2 Clinical Epidemiology Unit, Department of Clinical Sciences Lund, Orthopaedics, Lund University, Lund, Sweden; 3 Centre for Economic Demography, Lund University, Lund, Sweden; 4 Skåne University Hospital, Remissgatan 4, 221 85 Lund, Sweden

**Keywords:** Gender gap, Life expectancy, Decomposition, Avoidable deaths, UK, Mortality

## Abstract

**Background:**

In many high-income countries, life expectancy (LE) has increased, with women outliving men. This gender gap in LE (GGLE) has been explained with biological factors, healthy behaviours, health status, and sociodemographic characteristics, but little attention has been paid to the role of public health policies that include/affect these factors. This study aimed to assess the contributions of avoidable causes of death, as a measure of public health policies and healthcare quality impacts, to the GGLE and its temporal changes in the UK. We also estimated the contributions of avoidable causes of death into the gap in LE between countries in the UK.

**Methods:**

We obtained annual data on underlying causes of death by age and sex from the World Health Organization mortality database for the periods 2001–2003 and 2014–2016. We calculated LE at birth using abridged life tables. We applied Arriaga’s decomposition method to compute the age- and cause-specific contributions into the GGLE in each period and its changes between two periods as well as the cross-country gap in LE in the 2014–2016 period.

**Results:**

Avoidable causes had greater contributions than non-avoidable causes to the GGLE in both periods (62% in 2001–2003 and 54% in 2014–2016) in the UK. Among avoidable causes, ischaemic heart disease (IHD) followed by injuries had the greatest contributions to the GGLE in both periods. On average, the GGLE across the UK narrowed by about 1.0 year between 2001–2003 and 2014–2016 and three avoidable causes of IHD, lung cancer, and injuries accounted for about 0.8 years of this reduction. England & Wales had the greatest LE for both sexes in 2014–2016. Among avoidable causes, injuries in men and lung cancer in women had the largest contributions to the LE advantage in England & Wales compared to Northern Ireland, while drug-related deaths compared to Scotland in both sexes.

**Conclusion:**

With avoidable causes, particularly preventable deaths, substantially contributing to the gender and cross-country gaps in LE, our results suggest the need for behavioural changes by implementing targeted public health programmes, particularly targeting younger men from Scotland and Northern Ireland.

**Supplementary Information:**

The online version contains supplementary material available at 10.1186/s12963-021-00271-2.

## Introduction

Life expectancy (LE) has increased worldwide and steadily in developed countries, such as the UK [1]. This improvement draws special attention to their high-quality health systems regarding disease control, treatment, and prevention, among other population inherited characteristics [2]. An average citizen in the UK should live until the age of 81.3 years under the current age-specific death rates [3]. However, women outlive men. Even though this gender gap in LE (GGLE) has been declining over the last 20 years, there is still 3.7 years of difference in the GGLE observed in 2019 compared to 4.8 years in the early 2000s [4, 5]. Different explanations have been suggested for this GGLE including biological features (e.g. quantity of chromosomes, iron levels, and hormone disorders and triggers), behavioural (e.g. habits, lifestyle, and physical activity), health risk factors (e.g. alcohol consumption, smoking, obesity), and sociodemographic characteristics (e.g. risky jobs and sociocultural roles) [6, 7]. While men have been historically linked to more hazardous occupations (e.g. manual and elementary labours), the situation is changing with turndown of the mining and manufacturing industries together with an accelerated eradication of physical labours, as well as a reduced smoking prevalence among men [8, 9]. Simultaneously, women have increased their health risky behaviour (such as alcohol consumption, smoking, and obesity), reaching similar levels to that of men [8, 10]. All these patterns have likely contributed to the reduction in GGLE over the recent years in many countries around the world [11, 12].

Although the contribution of public health policies and quality healthcare aiming to enhance population health and reduce socioeconomic disparities plays a crucial role, less attention is given to their impact as whole in reducing the GGLE in the UK. The concept of avoidable mortality had been introduced as an indicator of the influences of healthcare quality and public health policies on population health [13]. Avoidable mortality considers all the deaths that could be avoided through the proper prevention and/or treatment. More specifically, the UK has established a long-term health plan called “Living well for longer” which considers national support for local action to decrease avoidable mortality over the life course [14].

A number of studies have shown a greater decline in the number of avoidable deaths over time than non-avoidable deaths globally [7, 15–20] which translated into significant contribution of avoidable death into gain in LE. Moreover, the decreases in avoidable deaths were generally larger in men than women leading to meaningful contributions of avoidable causes in reducing GGLE. For instance, avoidable causes of death were responsible for 1.4 years (78%) of reduction in the GGLE in Sweden between 1997 and 2018 [15]. Previous studies on the GGLE have shown that male LE is increasing in the UK while female LE remains constant and at lower levels than many comparable countries [5, 21–23]. However, to the best of our knowledge, no information is yet provided about the role of avoidable deaths into the GGLE and cross-country differences in LE in the UK. Cross-country differences allow to explore any potential differences in age- and cause-specific contributions to life expectancy among countries with very similar historical, cultural, and organizational background in addition to similarities in their healthcare systems. For instance, healthcare system is administered by the National Health Service (NHS). However, and since the devolution in the UK, there are some differences in the administration. A higher competition between the private and public sector in England where a patient-follows-the-money strategy was implemented, integration of health and social services primarily led by Northern Ireland, and free prescriptions and older adults care mainly in Scotland followed by Northern Ireland and Wales are examples of these differences [24]. All these variations might have impacted countries health performance and therefore country-specific avoidable mortality rate differently [24]. Indeed, remarkably higher life expectancy in England, particularly after the devolution, compared to Scotland might be attributed to these differences. Hence, cross-country comparison can provide crucial insights on the impacts of different policies taken over time, particularly when these policies seem to be growingly divergent.

Furthermore, maintaining quality of life and a healthy living while decreasing the gender gap in LE, essentially in older adults, are among the most important challenges of aging societies such as the UK and other high-income countries (HICs). Therefore, the present study aimed to analyse and compare the contributions of avoidable causes of death, as a measure of public health policies and healthcare quality impacts, into the GGLE and its changes in the UK between 2001–2003 and 2014–2016. In addition, we estimated the contributions of avoidable causes of death into the gap in LE between countries in the UK.

## Methods

### Data and analytical sample

This research focuses on the UK as a whole and its countries (i.e. England & Wales, Scotland, and Northern Ireland). We obtained annual data on underlying causes of death by age and sex from the World Health Organization (WHO) mortality database using two timeframes: 2001–2003 and 2014–2016 [25]. The civil registration coverage of cause-of-death data was 99.6% and 100% for the periods analysed, respectively [26, 27]. We aggregate the data over 3 years to avoid the potential effect of fluctuation in number of deaths in a single year especially for within country analyses where the number of deaths for some causes can be small. These data contain country-level information on the number and cause of deaths classified by gender, age-group (< 1, 1–4, 5–9, 10–14, …, 85 +), and underlying cause of death submitted annually by WHO member states from their civil registration systems, including only medically certified deaths. The underlying cause of death is described as the injury or disease which has begun the sequence of morbid incidents leading to death or the situation causing the accident that therefore produced the fatal injury. We also obtained the data on population by sex, age, and year from the same source. Then, we classified causes of death according to the recently developed list of avoidable mortality from the Organisation for Economic Co-operation and Development (OECD) and the statistical office of the European Union (Eurostat) [28]. This list is used by the Office of National Statistics (ONS) in the UK [29]. We divided causes of death into five mutually exclusive categories: (1) only treatable (or amenable), (2) only preventable, (3) treatable and preventable, (4) ischaemic heart disease (IHD), and (5) non-avoidable. It should be noted that while IHD is a treatable and preventable cause, we analysed it separately due to large number of IHD deaths which might mask the contributions of other causes. Treatable causes are those which could have been avoided through effective and timely healthcare interventions or secondary prevention and treatment. Treatable and preventable causes are those which could have been avoided through a combination of public health prevention and healthcare interventions (they have been allocated equally when no strong evidence of predominance is available). We further divide categories (1) to (3) into subcategories. Preventable causes are those which could have been avoided through primary or public health prevention interventions [28]. Treatable and preventable causes included cerebrovascular diseases, diabetes mellitus, hypertensive diseases, and other causes. Only preventable causes included alcohol-related deaths, lung cancer, other preventable cancers, drug-related deaths, infectious diseases, injuries, and other causes. Only treatable causes included cancer, digestive system diseases, genitourinary system diseases, infectious disease, pregnancy, childbirth and perinatal period diseases, respiratory system diseases, and other causes (full details of these causes and their respective ICD-10 codes can be found in Additional file 1). It should be noted that there is an upper age limit of 74 years for avoidable causes, meaning that deaths from avoidable causes among those aged ≥ 75 years are considered as non-avoidable.

### Statistical analyses

We calculated gender-specific LE at birth for the UK and its countries using abridged life tables for both study's periods [30].

The GGLE was computed as the difference in LE between women and men (i.e. women LE minus men LE). The GGLE in each period and its changes between two periods were decomposed by age group and cause of death using Arriaga’s decomposition method [31, 32]. For interpretation purposes, a negative (positive) contribution to the GGLE over a single period indicates disadvantage (advantage) in LE in women over men. On the other hand, when investigating changes in the GGLE between two periods, a positive (negative) value reflect widening (narrowing) the GGLE over time. 

Additionally, we ran a secondary analysis to decompose the cross-country gap in LE by age group and cause of death in the 2014–2016 period. The analysis was employed to quantify the differences between countries to see the impact of public health policies and healthcare quality in each country and to help tailoring context specific public health policies and interventions. To do so, we selected the England & Wales with the highest LE for both men and women as the benchmark and subtracted Scotland and Northern Ireland LE values from England & Wales. In this analysis, a positive value reflects the contribution into LE advantage in England and Wales over the comparator country, while the opposite is the case for a negative value. It should be mentioned that in the WHO mortality database the data for England & Wales are presented together and this is the reason why we present the results for these two countries together.

All data analyses were computed using Stata version 16 and Microsoft Excel version 16.48 2021.

## Results

There were 483,186 deaths due to avoidable causes (103,701 treatable causes, 192,153 preventable causes, 65,566 treatable and preventable causes, and 127,766 IHD) in the UK during 2001–2003 (in Additional file 2: Table A1). The corresponding figure in 2014–2016 was 404,660 deaths (93,853 treatable causes, 201,122 preventable causes, 41,947 treatable and preventable causes, and 67,738 IHD). The absolute number of non-avoidable causes deaths rose from 1,341,332 to 1,365,666 between these two periods. Between 2001–2003 and 2014–2016, the proportion of avoidable causes from all-cause death among men declined from 34.4 to 28.2% in the UK, from 33.5 to 27.7% in England & Wales, from 37.4 to 31.4% in Northern Ireland, and from 41.4 to 32.8% in Scotland (Additional file 3). Corresponding reductions among women were from 19.4 to 17.7% in the UK, from 18.8 to 17.3% in England & Wales, from 21.5 to 19.2% in Northern Ireland, and from 23.8 to 20.9% in Scotland. Across avoidable causes subgroups, while the proportions from all-cause death declined for treatable causes, treatable and preventable causes, and IHD in both sexes in all countries, for preventable causes it only declined among men in Scotland (from 19.8% in 2001–2003 to 18.3% in 2014–2016). On the contrary, proportion of preventable causes from all-cause death in UK rose from 6.7 to 8.1% among women and was stable at 14.8% among men.

### Gender gap in life expectancy

Table 1 depicts how LE and the GGLE has changed over the last decade within the UK. LE in men increased by 3.4 years in the UK between 2001–2003 to 2014–2016, compared to 2.5 years gain in women. On average, the GGLE narrowed by about 1-year in the UK (ranged from 0.9 years in England & Wales to 1.3 years in Scotland).

**Table 1:** Life expectancy and gender gap in life expectancy across the UK in 2001–2003 and 2014–2016

Country	Gender	2001–2003		2014–2016		Gain in LE between 2001–2003 and 2014–2016, years
		LE, years	GGLE, years	LE, years	GGLE, years	
UK	Women	80.47	4.60	82.93	3.64	2.46
	Men	75.87		79.29		3.42
England & Wales	Women	80.64	4.60	83.12	3.60	2.48
	Men	76.12		79.52		3.40
Northern Ireland	Women	80.46	4.86	82.40	3.76	1.94
	Men	75.59		78.64		3.05
Scotland	Women	78.84	5.34	81.19	4.04	2.35
	Men	73.50		77.15		3.65

*GGLE*: Gender gap in life expectancy; *LE*: Life expectancy

**Fig. 1:**
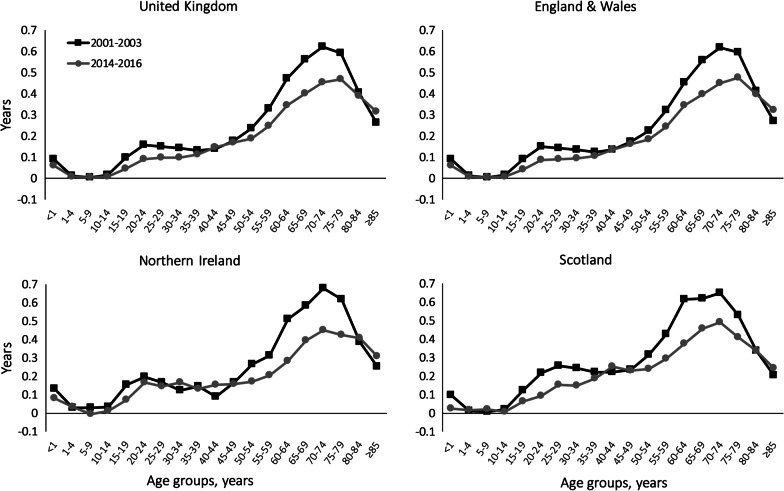
Age-specific contributions to the gender gap in life expectancy across the UK in 2001–2003 and 2014–2016. Positive (negative) values indicate life expectancy advantage (disadvantage) in women

In the UK, the age groups 70–74 years and 75–79 years had the greatest contributions into the GGLE in 2001–2003 and 2014–2016, respectively (Fig. 1 and in Additional file 2: Table A2). Similar pattern was seen in England & Wales, while in Scotland and Northern Ireland, the age group 70–74 years had the largest contributions into the GGLE in both periods.

**Fig. 2:**
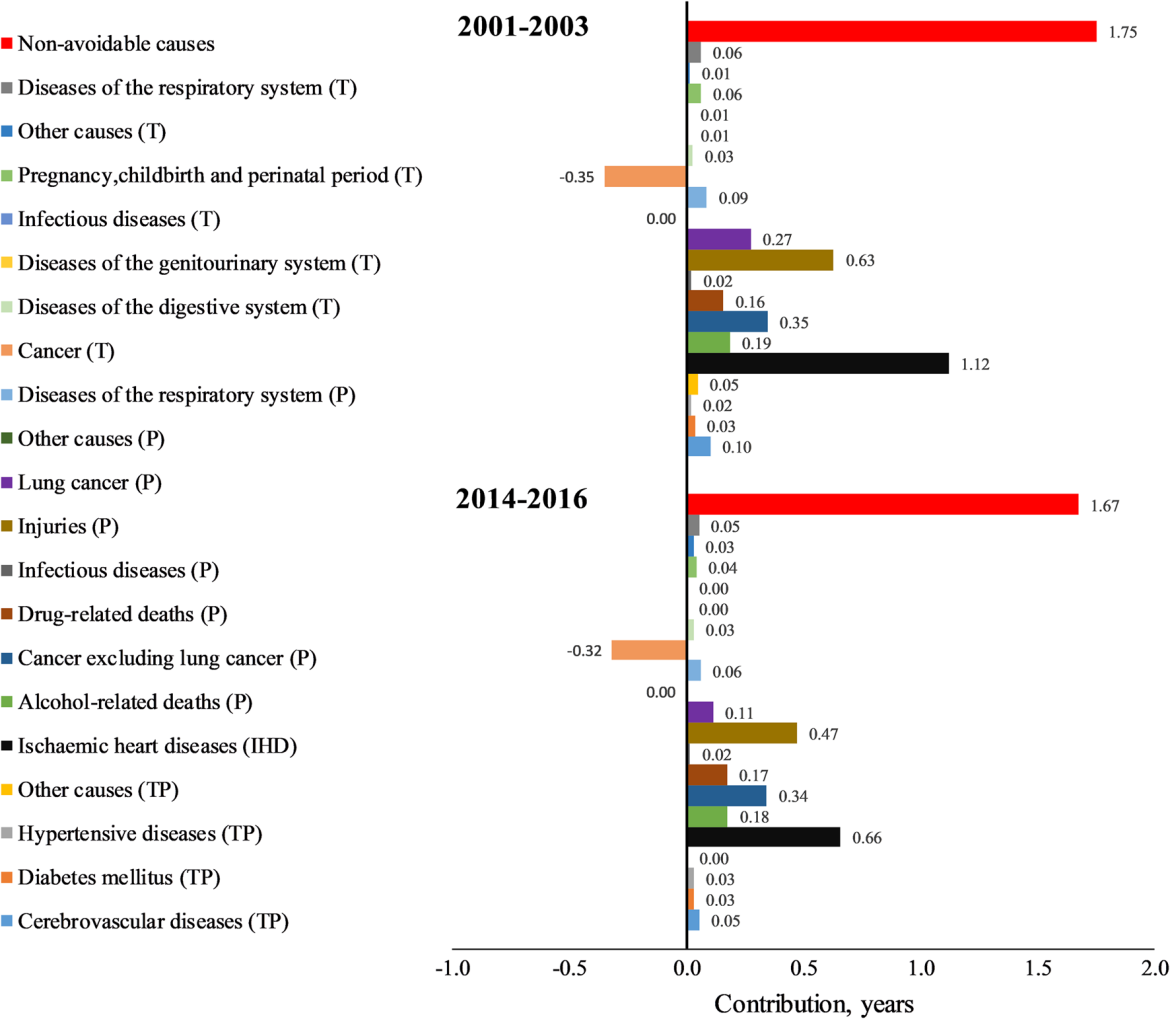
The contributions of the causes of death to the gender gap in life expectancy in the UK in 2001–2003 and 2014–2016. T: Treatable, P: preventable, TP: treatable and preventable

Avoidable causes contributed to 2.9 years of the GGLE in 2001–2003 and this declined to 2 years in 2014–2016 (Fig. 2, in Additional file 2: Table A2). Among avoidable causes, IHD followed by injuries had the greatest contributions to the GGLE in both periods. Similar patterns were seen across the countries with only exception in Northern Ireland for 2014–2016 where injuries were the leading contributor to the GGLE (Additional file 2: Table A3–A5). Among avoidable causes, “treatable cancers” and “other preventable causes” were the only groups with negative contributions to the GGLE in both periods (LE disadvantage in women), even though the latter group contributions were negligible.

**Fig. 3:**
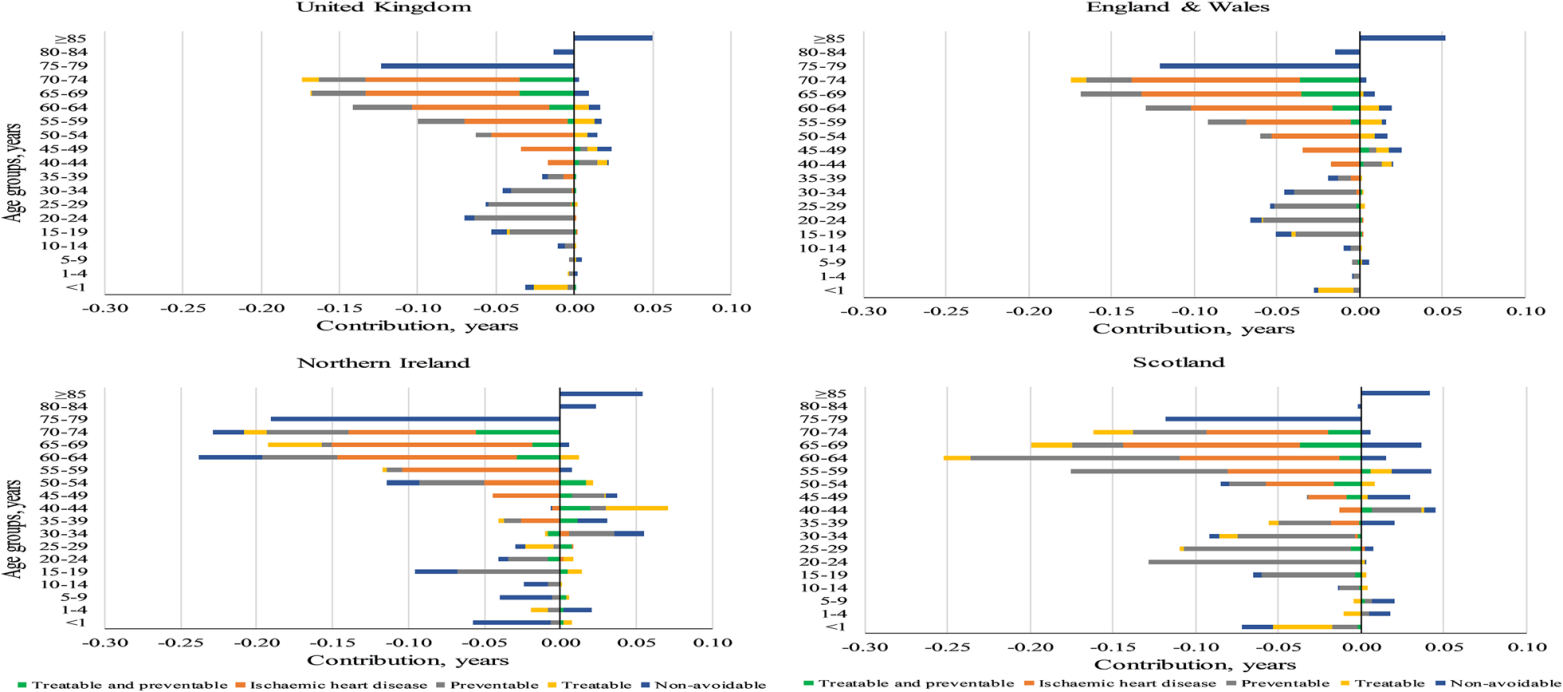
Age- and cause-specific contributions to the change in gender gap in life expectancy between 2001–2003 and 2014–2016 across the UK. Positive (negative) values indicate widening (narrowing) the gender gap in life expectancy

The age group 70–74 years had the greatest contribution (0.17 years) into the reduction in the GGLE in the UK (Fig. 3). In addition, age groups 5–9 years, 40–44 years, and 85 + years contributed to widening the GGLE in the UK. While these patterns were generally similar across countries, there were some variations (e.g. age groups 30–34 years and 80–84 years contributed to narrow the GGLE in England & Wales and Scotland while opposite was seen in Northern Ireland). Avoidable causes accounted for 0.9 years (out of 1 year) of the narrowing GGLE in the UK (Additional file 2: Table A2). Among avoidable causes, IHD followed by two preventable causes (i.e. lung cancer and injuries) had the highest contributions to narrowing the GGLE (0.8 out of 1 year in the UK, 0.8 out of 0.9 years in England & Wales, 0.9 out of 1.1 years in Northern Ireland, and 1 out of 1.3 years in Scotland; see Additional file 2: Table A2-A5). While in England & Wales and Northern Ireland, IHD had greater contributions into the narrowing GGLE from preventable causes combined, the opposite was observed in Scotland (see Table 2 and Additional file 2: Table A3-A5). On the other hand, treatable causes widened the GGLE in England & Wales while narrowed it in Northern Ireland and Scotland. Moreover, non-avoidable causes widened the GGLE in Scotland (0.05 years) while narrowed it in England & Wales (0.08 years) and Northern Ireland (0.26 years; see Additional file 2: Table A3-A5).

**Table 2:** Cause-specific contributions (years) to changes in the gender gap in life expectancy across the UK between 2001–2003 and 2014–2016

Country/cause of death	Avoidable				Non-avoidable	Total
	Treatable and preventable	IHD	Preventable	Treatable		
United Kingdom	−0.08	−0.46	−0.35	0.01	−0.08	−0.96
England & Wales	−0.08	−0.46	−0.31	0.02	−0.08	−0.92
Northern Ireland	−0.04	−0.56	−0.24	−0.01	−0.26	−1.10
Scotland	−0.10	−0.45	−0.69	−0.10	0.05	−1.29

*IHD*: Ischaemic heart diseases. More detailed information can be found in Additional file 2: Table A2-A5

### Cross-country gap in life expectancy

**Fig. 4:**
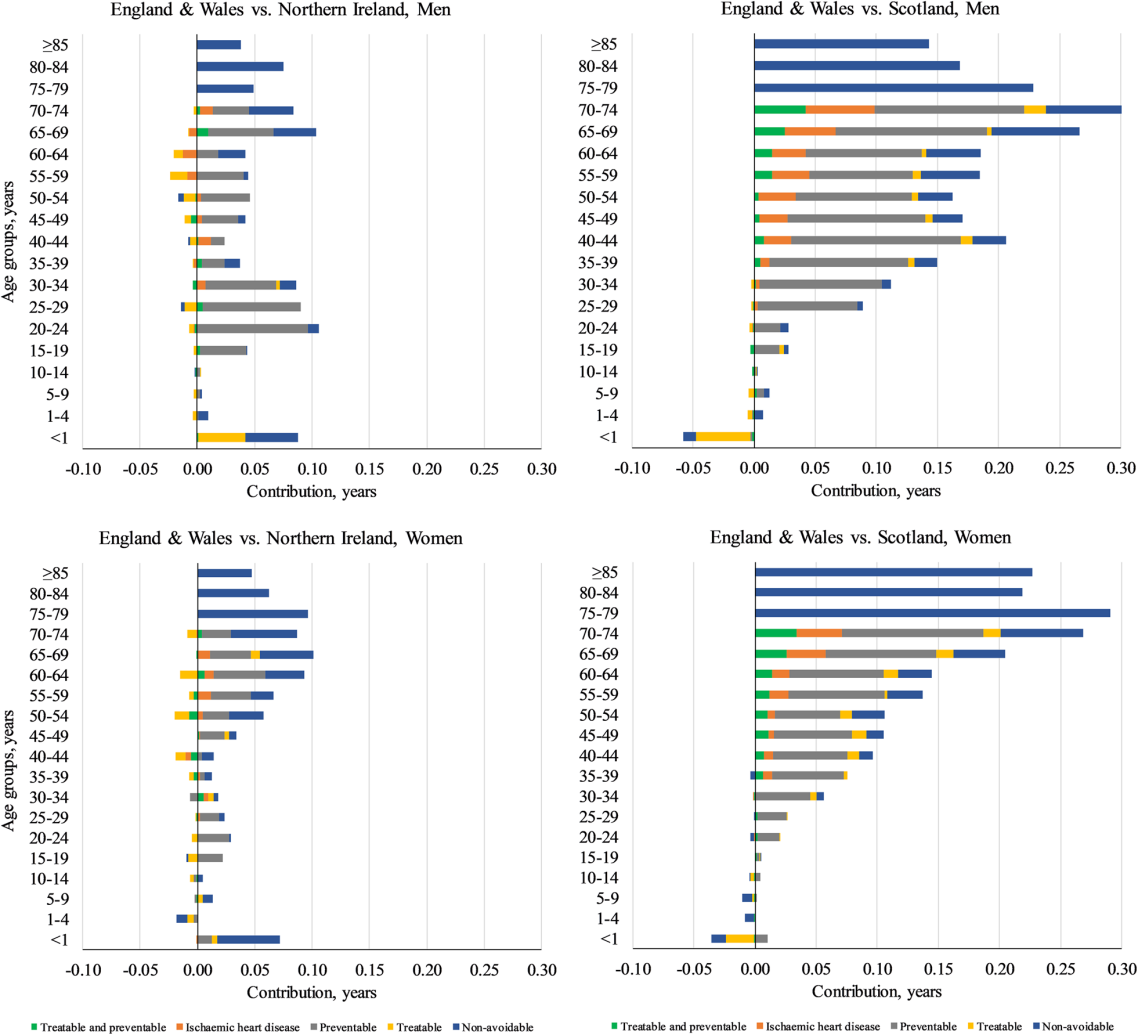
Age- and cause-specific contributions to the gap in life expectancy between England & Wales and the rest of the UK. Positive (negative) values indicate life expectancy advantage (disadvantage) in England & Wales

During 2014–2016, LE for men in England & Wales was about 0.9 and 2.4 years longer than those in Northern Ireland and Scotland, respectively, and age groups < 50 years accounted for 0.5 and 0.7 years of these gaps (Fig. 4, panel A). Among women, the LE gaps were 0.7 years with Northern Ireland and 1.9 years with Scotland, with those aged < 50 years accounting for 0.2 and 0.3 years of these gaps, respectively (Fig. 4, panel B, Additional file 2: Table A6–A7). Avoidable causes were responsible for about 0.5 out of 0.9 years LE gap among men when comparing England and Wales with Northern Ireland, with three preventable causes of injuries (0.3 years), alcohol-related deaths (0.1 years), and lung cancer (0.1 years) as the leading contributors. For women, contribution of avoidable causes to the England & Wales vs. Northern Ireland LE gap was lower (0.2 out of 0.7 years), with two preventable causes of lung cancer and alcohol-related deaths followed by treatable cancers (about 0.1 years each) representing the highest contributions. On the other hands, avoidable causes contributed to around 1.5 years (out of 2.4 years) and 1 year (out of 1.9 years) of the LE gap between England & Wales and Scotland among men and women, respectively, with preventable drug-related deaths representing the highest contributions among avoidable causes in both sexes. Noticeably, mortality rate among children aged < 10 years was lower in Scotland compared to England & Wales, particularly in < 1-year age group from treatable cause of pregnancy, childbirth, and perinatal deaths (Additional file 4).

## Discussion

In this study, we quantified the potential contributions of public health policies and healthcare quality, operationalized as avoidable deaths, on the gender and cross-country gaps in LE in the UK. We found a GGLE of 3.6 years in 2014–2016 representing about 1-year decline since 2001–2003. Moreover, men and women in England &Wales had longer LE than their counterparts in Northern Ireland and Scotland. Avoidable causes accounted for a substantial portion of these patterns, with preventable causes (specifically lung cancer and injuries) having the greatest contributions to the gender and cross-country gaps in LE, while IHD had the highest contributions to narrowing GGLE over time.

Our results are in line with the previous findings in the UK and other locations [5–7, 15, 16, 21, 33, 34]. We observed significant gain in LE in the UK over the study period largely due to reduction in deaths from avoidable causes such as IHD, lung cancer, injuries, and lifestyle diseases (e.g. smoking, alcohol consumption). These declines in avoidable mortality resulted in a decrease over the decade in the contribution of avoidable deaths to the GGLE (from 2.9 years to 2.0 years) which is consistent with previous literature [7, 15–20]. These improvements can partially be attributed to public health policies, such as those mentioned below, implemented during the period observed [35]. First, the smoking ban launched in 2007 in the UK has made strides in decreasing smoking rates while enhancing tobacco control and bringing down advertising [36, 37]. Second, the traffic-light labelling on food to display nutritional information, the junk food advertising ban during children TV times in 2007, and doors food hygiene scores to comply with local food regulations (for every food business) aiming to tackle obesity and food-borne diseases altogether [38, 39]. Third, vaccination programmes include childhood flu vaccine (which pilots began in 2012) and the introduction of the human papillomavirus (HPV) in children since 2008 [40, 41]. Fourth, the cancer screening improvements started off in England (2006), Scotland (2007), Wales (2008), and Northern Ireland (2010), to offer free screenings to people older than 50 years of age [42]. Other policies have included drug testing and minimum drink price to reduce drug and alcohol consumption [43].

Overall, LE in the UK has stalled since 2010, showing the lowest increase rate compared to other HICs [5, 21–23]. LE among men has slowly increased while it has remained constant for women [22]. This may be explained to some extent by different ex-ante attitudes towards health between males and females. For example, men seek less healthcare than women [44, 45]; therefore, any behavioural health policy is likely to be more effective on males who simply have more room for improvement. Moreover, while IHD’s contribution to GGLE has declined across the UK, the role of drug-related deaths rose. Drug-related deaths belong to the “deaths of despair” [46], which are attributable to socio-economic conditions rather than healthcare policies and have been increasing also in the UK [47]. The slowdown in LE growth may be attributed to other than winter-related mortality in the UK (e.g. caused by influenza and other respiratory diseases during winter), which was the leading cause since the early 90s. Mortality has increased, especially for those aged 40–49 and older than 80 years, mainly due to suicide, drug, and alcohol misuse. Mortality is decreasing significantly for people aged 70–75, but it remains almost steady for those older than this age segment [48]. We infer that social and economic conditions have undermined health at these age groups. For instance, preventable mortality most concentrated among the poorest, and this might be occurring due to existing social, economic, and financial disparities within the regions. The 2020 Marmot review about social inequalities in England [43] has highlighted a shorter life expectancy in regions with high proportion of deprived areas throughout the UK, where gradient is steeper over the last decade and especially among women in the poorest 10% whose LE has declined since 2010. Moreover, at the same period, healthy LE has declined, particularly for women, indicating increases in number of years spent in poor health for both women and men [43].

The observed decline in the GGLE in our study, particularly large contributions from preventable causes and IHD, possibly imply declining the gender gap in behavioural risk factors such as smoking, alcohol use, and obesity [5, 7, 15, 16, 33, 34]. For example, in recent years women smoked at the same rate as men [8, 11] and the gender gap in alcohol consumption has been narrowed considerably [49, 50]. Both these risk factors affect women disproportionally compared with men, including 25% higher risk of heart failure due to smoking in women than men [11] or more significant risks of brain damage, heart muscle, cancers, and liver disease from alcohol consumption in women [51]. Moreover, obesity in the UK has remained slightly higher for women than men (30% vs 27%) [10]. Furthermore, men have benefited from less physically demanding work over the past few decades and higher incomes, which might have contributed to the narrowed GGLE [52]. Indeed, the ONS reported that based on death patterns in 2015, LE in professional men in the UK might surpass LE in women [9]. Additionally, the age distribution of deaths has been historically less scattered for women than men, implying that the reduction in GGLE might be a consequence of a change in mortality's age pattern [53]. The age pattern of mortality is reflected by the high reduction in IHD deaths in older men throughout the UK, contributing largely to narrowing the GGLE. It should be noted that despite the favourable behavioural changes in men over time, still women experience LE advantage from many of avoidable causes over men, meaning that more changes among men are needed to further narrow the GGLE in the UK.

Our cross-country comparison revealed that injuries as well as drug- and alcohol-related deaths contributed substantially to LE disadvantages in Northern Ireland and Scotland versus England & Wales. Cohort studies from Scotland [54, 55] have evidenced that alcohol and drug consumption, and consecutive related deaths, increased sharply during the 1990s due to the massive wave of men living in deprived areas arguably as a consequence of political and economic policies [54, 56]. There is an ageing profile of problem drug users impacting LE estimates over time, representing a period effect, and especially in men. This suggests that further locality-specific interventions to prevent the diseases with the highest local burden are necessary to improve LE and GGLE. For all countries, policies oriented to tackle avoidable deaths through extensive treatment and prevention of diseases related to excessive alcohol consumption and tobacco, infectious diseases, and chronic conditions are crucial to strengthening the health system's quality to improve LE and ensure a better health status [57]. Specifically, in Scotland, policies targeting drug users’ ageing, and their respective co-morbidities, would need to be adapted to reduce the cross-country gap in LE and narrow the GGLE.

We should acknowledge that our study has a number of limitations. First, comparability with existing literature is not direct as the OECD classification of causes of death used has varied and adjusted over time. Second, the OECD classification uses a common age threshold of 75 years to define premature deaths, but this might not reflect all countries’ population characteristics. In particular, this age threshold may underestimate the general number of deaths that could be avoided through better health care and prevention for people aged 75 and older which are a substantial proportion of the UK’s total population. This threshold should be redefined in the future to account for gains in LE and according to each country’s demographics. Third, avoidable mortality is not a direct measure of the health system’s performance and capacity, even though it indirectly assesses whether public health and healthcare policies in place are avoiding deaths caused by treatable or preventable causes over time. Also, it does not include diseases’ prevalence and severity nor the effect of policies on quality of life, but it might be descriptively associated to the policies from an epidemiological non-causal perspective. Fourth, the analysis is time sensitive as the introduction of new technologies might change the results and course of the leading causes of death. Fifth, mortality is not always an accurate indicator to measure health quality; other characteristics such as population health comorbidities should be considered in the future. Sixth, the Arriaga approach may underestimate the causes of death occurring at older ages; however, it is a reliable tool to estimate the direct and indirect effects of health policies on mortality rates and LE within specific age groups [20]. Also, compared to other decomposition methods, this approach is clear and accessible to apply to traditional life table data, especially when data are given in discrete time [32, 58]. It should be also noted that results of different decomposition methods are generally similar [32]. Seventh, we used the OECD classification [28] which is slightly different to previously used standards in the UK and hence limit cross-study comparisons [29]. For instance, some chronic illnesses (bladder, breast, and skin cancers) changed from treatable to preventable. At the same time, a few infectious diseases (influenza and HIV/AIDS) were modified from treatable and preventable to only the latter. These differences should be taken into consideration when interpreting our findings.

Finally, the selection of avoidable mortality relies on the relative effectiveness of public health and healthcare policies /programmes that may avert and halt deaths, operating from the healthcare system backwards. Avoidable conditions are expected to be averted even after the disease is developed (e.g. tuberculosis, cervical cancer, etc.), or prevented from occurring (e.g. lung cancer attributed to smoking or liver cirrhosis due to alcohol consumption). It is arduous to evaluate impact of specific health programmes or policies on overall population health because the reduction in mortality for heart diseases (treatable), for instance, might be attributed to either change in lifestyles or food habits, which are contrarily classified as “preventable”. The same occurs with transport and accidental injuries (preventable), which are impacted by the enhancement of emergency services and infrastructure, which might be conversely defined as “treatable”. We suggest that further research needs to be done for a detailed comprehension of specific causes of death and how they are impacted. However, timely medical interventions, including secondary prevention (effective treatment and early detection), are beneficial in averting deaths.

## Conclusions

This study highlights a reduction in the contribution of avoidable mortality to the GGLE in the UK over the last decade. There was a significant contribution of avoidable causes, especially preventable and IHD, into rising LE among men, which contributed into narrowing the GGLE. The results suggest the need for further improvements in preventive and treatment measures by implementing public health and healthcare policies and programmes targeting at avoidable deaths such as ischaemic heart disease, alcohol- and drug-related deaths, cancer, and injuries, specifically for younger men from Scotland and Northern Ireland.

## Supplementary Information

**Additional file 1.** List of avoidable causes of death according to the Organisation for Economic Co-operation and Development and the statistical office of the European Union.

**Additional file 2:** **Table A1.** Absolute number of avoidable and non-avoidable causes of death in the UK in 2001-2003 and 2014-2016, stratified by sex. **Table A2.** Results of Arriaga’s decomposition for the UK.** Table A3. **Results of Arriaga’s decomposition for England & Wales. **Table A4. **Results of Arriaga’s decomposition for Northern Ireland. **Table A5.** Results of Arriaga’s decomposition for Scotland. **Table A6.** Age- and cause-specific contributions to gap in life expectancy between England & Wales vs. Northern Ireland. **Table A7.** Age- and cause-specific contributions to gap in life expectancy between England & Wales vs. Scotland.

**Additional file 3.** Proportion of avoidable causes from all-cause death across the UK, by sex.

**Additional file 4.** Age-specific contributions to the gap in life expectancy between England & Wales and the rest of the UK, by sex. Positive (negative) values indicate life expectancy advantage (disadvantage) in England & Wales.

## Data Availability

Data are shared publicly at either the WHO or EUROSTAT websites.

## Abbreviations

EUROSTAT: Statistical Office of the European Union**;** GGLE: Gender Gap in Life Expectancy
ICD: International Classification of Diseases and Related Health Problems
IHD: Ischaemic Heart Disease
LE: Life Expectancy
OECD: Organisation for Economic Co-operation and Development
ONS: Office of National Statistics in the UK
SDGs: Sustainable Development Goals
UK: United Kingdom
UN: United Nations
WHO: World Health Organization
