# Supplementary material for: The contributions of public health policies and healthcare quality to gender gap and country differences in life expectancy in the UK
Source: Popul Health Metr. 2021 Oct 20;19:40. doi: 10.1186/s12963-021-00271-2 (PMC8527782; doi:10.1186/s12963-021-00271-2)
Supplement: Supplementary file 1 — Additional file 1. List of avoidable causes of death according to the Organisation for Economic Co-operation and Development and the statistical office of the European Union. [file 12963_2021_271_MOESM1_ESM.pdf]

Additional file 1. List of avoidable causes of death according to the Organisation for Economic Co-operation and Development and the statistical office of the European Union.

Condition group and cause	ICD-10 codes	Age	Treatable	Preventable
**Infectious diseases**				
Intestinal diseases	A00-A09	0-74		•
Diphtheria, Tetanus, Poliomyelitis	A35, A36, A80	0-74		•
Whooping cough	A37	0-74		•
Meningococcal infection	A39	0-74		•
Sepsis due to streptococcus pneumonia and sepsis due to haemophilus influenza	A40.3, A41.3	0-74		•
Haemophilus influenza infections	A49.2	0-74		•
Sexually transmitted infections (except HIV/AIDS)	A50-A60, A63, A64	0-74		•
Varicella	B01	0-74		•
Measles	B05	0-74		•
Rubella	B06	0-74		•
Viral Hepatitis	B15-B19	0-74		•
HIV/AIDS	B20-B24	0-74		•
Malaria	B50-B54	0-74		•
Haemophilus and pneumococcal meningitis	G00.0, G00.1	0-74		•
Tuberculosis	A15-A19, B90, J65	0-74	• (50%)	• (50%)
Scarlet fever	A38	0-74	•	
Sepsis	A40 (excl. A40.3), A41 (excl. A41.3)	0-74	•	
Cellulitis	A46, L03	0-74	•	
Legionnaires disease	A48.1	0-74	•	

Streptococcal and enterococci infection	A49.1	0-74	•	
Other meningitis	G00.2, G00.3, G00.8, G00.9	0-74	•	
Meningitis due to other and unspecified causes*	G03	0-74	•	
Neoplasms				
Lip, oral cavity and pharynx cancer	C00-C14	0-74		•
Oesophageal cancer	C15	0-74		•
Stomach cancer	C16	0-74		•
Liver cancer	C22	0-74		•
Lung cancer	C33-C34	0-74		•
Mesothelioma	C45	0-74		•
Skin (melanoma) cancer	C43	0-74		•
Bladder cancer	C67	0-74		•
Cervical cancer	C53	0-74	• (50%)	• (50%)
Colorectal cancer	C18-C21	0-74	•	
Breast cancer (female only)	C50	0-74	•	
Uterus cancer	C54, C55	0-74	•	
Testicular cancer	C62	0-74	•	
Thyroid cancer	C73	0-74	•	
Hodgkin's disease	C81	0-74	•	
Lymphoid leukaemia	C91.0, C91.1	0-74	•	
Benign neoplasm	D10-D36	0-74	•	
Endocrine and metabolic diseases				
Nutritional deficiency anaemia	D50-D53	0-74		•
Diabetes mellitus	E10-E14	0-74	• (50%)	• (50%)

Thyroid disorders	E00-E07	0-74	•	
Adrenal disorders	E24-E25 (except E24.4), E27	0-74	•	
**Diseases of the nervous system**				
Epilepsy	G40, G41	0-74	•	
**Diseases of the circulatory system**				
Aortic aneurysm	I71	0-74	• (50%)	• (50%)
Hypertensive diseases	I10-I13, I15	0-74	• (50%)	• (50%)
Ischaemic heart diseases	I20-I25	0-74	• (50%)	• (50%)
Cerebrovascular diseases	I60-I69	0-74	• (50%)	• (50%)
Other atherosclerosis	I70, I73.9	0-74	• (50%)	• (50%)
Rheumatic and other heart diseases	I00-I09	0-74	•	
Venous thromboembolism	I26, I80	0-74	•	
**Diseases of the respiratory system**				
Influenza	J09-J11	0-74		•
Pneumonia due to streptococcus pneumonia or haemophilus influenza **	J13-J14	0-74		•
Chronic lower respiratory diseases	J40-J44	0-74		•
Lung diseases due to external agents	J60-J64, J66-J70, J82, J92	0-74		•
Upper respiratory infections	J00-J06, J30-J39	0-74	•	
Pneumonia, not elsewhere classified or organism unspecified	J12, J15, J16-J18	0-74	•	
Acute lower respiratory infections	J20-J22	0-74	•	
Asthma and bronchiectasis	J45-J47	0-74	•	
Adult respiratory distress syndrome	J80	0-74	•	

Pulmonary oedema	J81	0-74	•
Abscess of lung and mediastinum pyothorax	J85, J86	0-74	•
Other pleural disorders	J90, J93, J94	0-74	•
Diseases of the digestive system			
Gastric and duodenal ulcer	K25-K28	0-74	•
Appendicitis	K35-K38	0-74	•
Abdominal hernia	K40-K46	0-74	•
Cholelithiasis and cholecystitis	K80-K81	0-74	•
Other diseases of gallbladder or biliary tract	K82-K83	0-74	•
Acute pancreatitis	K85.0, K85.1, K85.3, K85.8, K85.9	0-74	•
Other diseases of pancreas	K86.1, K86.2, K86.3, K86.8, K86.9	0-74	•
Diseases of the genitourinary system			
Nephritis and nephrosis	N00-N07	0-74	•
Obstructive uropathy	N13, N20-N21, N35	0-74	•
Renal failure	N17-N19	0-74	•
Renal colic	N23	0-74	•
Disorders resulting from renal tubular dysfunction	N25	0-74	•
Unspecified contracted kidney, small kidney of unknown cause	N26-N27	0-74	•
Inflammatory diseases of genitourinary system	N34.1, N70-N73, N75.0, N75.1, N76.4, N76.6	0-74	•

Prostatic hyperplasia	N40	0-74	•	
**Pregnancy, childbirth and the perinatal period**				
Tetanus neonatorum	A33	0-74		•
Obstetrical tetanus	A34	0-74		•
Pregnancy, childbirth and the puerperium	O00-O99	0-74	•	
Certain conditions originating in the perinatal period	P00-P96	0-74	•	
**Congenital malformations**				
Certain congenital malformations (neural tube defects)	Q00, Q01, Q05	0-74		•
Congenital malformations of the circulatory system (heart defects)	Q20-Q28	0-74	•	
**Adverse effects of medical and surgical care**				
Drugs, medicaments and biological substances causing adverse effects in therapeutic use	Y40-Y59	0-74	•	
Misadventures to patients during surgical and medical care	Y60-Y69, Y83-Y84	0-74	•	
Medical devices associated with adverse incidents in diagnostic and therapeutic use	Y70–Y82	0-74	•	
**Injuries**				
Transport Accidents	V01-V99	0-74		•
Accidental Injuries	W00-X39, X46-X59	0-74		•
Intentional self-harm	X66-X84	0-74		•
Event of undetermined intent	Y16-Y34	0-74		•
Assault	X86-Y09, U50.9	0-74		•

Alcohol-related and drug-related deaths***			
Alcohol-specific disorders and poisonings	E24.4, F10, G31.2, G62.1, G72.1, I42.6, K29.2, K70, K85.2, K86.0, Q86.0, R78.0, X45, X65, Y15	0-74	•
Other alcohol-related disorders	K73, K74.0-K74.2, K74.6-K74.9	0-74	•
Drug disorders and poisonings	F11-F16, F18-F19, X40-X44, X85, Y10-Y14	0-74	•
Intentional self-poisoning by drugs	X60-X64	0-74	•

Notes: More information can be found elsewhere; OECD 2019 (http://www.oecd.org/health/health-systems/Avoidable-mortality-2019-Joint-OECD-Eurostat-List-preventable-treatable-causes-of-death.pdf). Full definitions in Table B1.

*ICD-10 code J02 (Acute pharyngitis) was included in both infectious diseases and diseases of the respiratory system broad cause groupings. To avoid double counting, J02 has been removed from infectious diseases. This has not affected the measures of overall avoidable, preventable and treatable mortality, but will have a minimal impact on infectious diseases broad cause groupings.

** OECD, in their final deliberations, removed these codes from infectious diseases to diseases of the respiratory system. This has not affected the measures of overall avoidable, preventable and treatable mortality, but has a small impact on the diseases of the respiratory system and infectious diseases broad cause groupings.

*** Additional breakdowns in the alcohol-related and drug-related deaths broad cause groupings have been included to ensure clarity for users. It should be noted that drug-related deaths include both illegal and legal drugs. These changes have not impacted the ICD-10 codes included in this grouping nor the findings.
