# Supplementary figures and images for: The contributions of public health policies and healthcare quality to gender gap and country differences in life expectancy in the UK
Source: Popul Health Metr. 2021 Oct 20;19:40. doi: 10.1186/s12963-021-00271-2 (PMC8527782; doi:10.1186/s12963-021-00271-2)

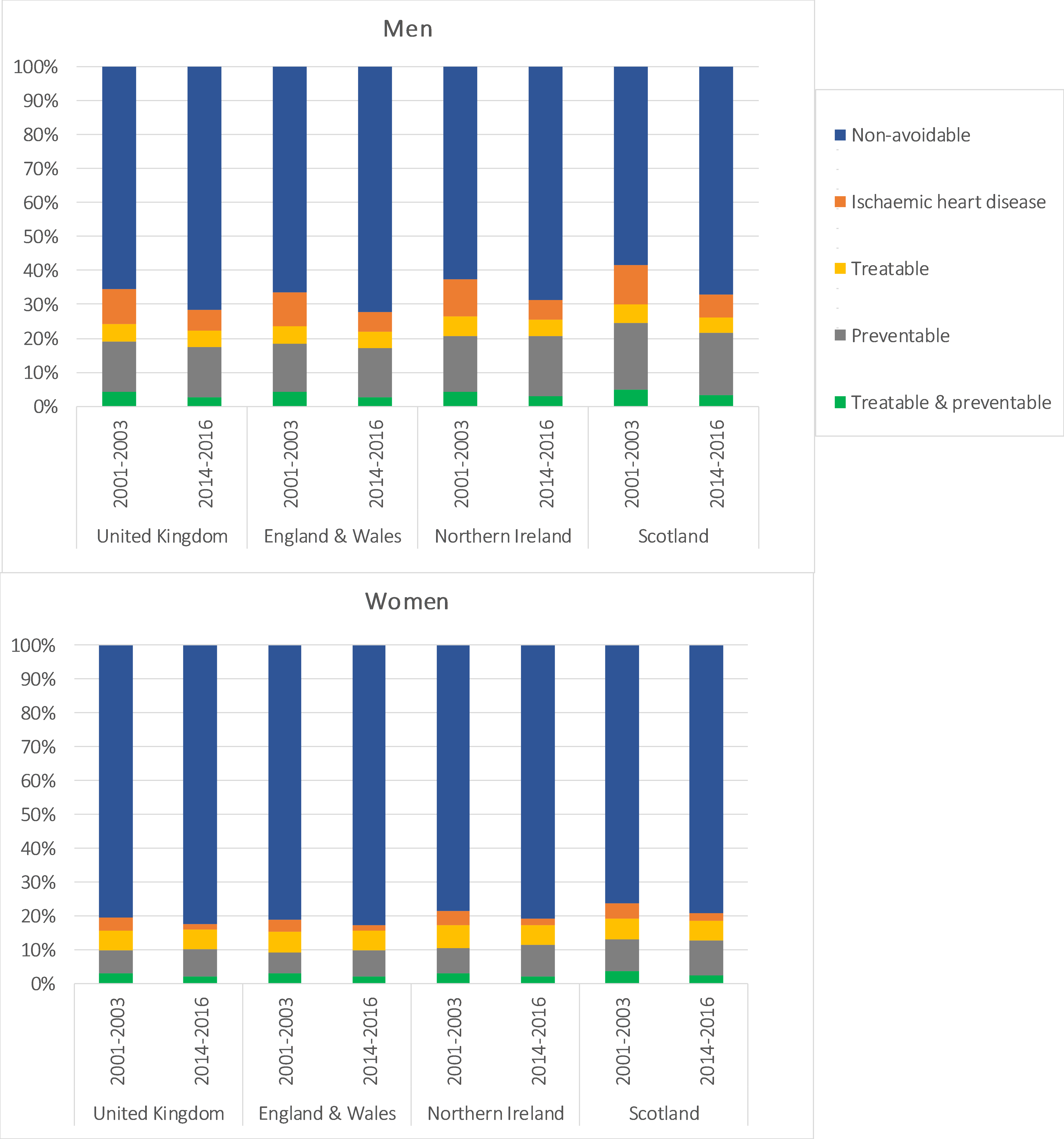

Supplement: Supplementary file 3 — Additional file 3. Proportion of avoidable causes from all-cause death across the UK, by sex. [file 12963_2021_271_MOESM3_ESM.tif]

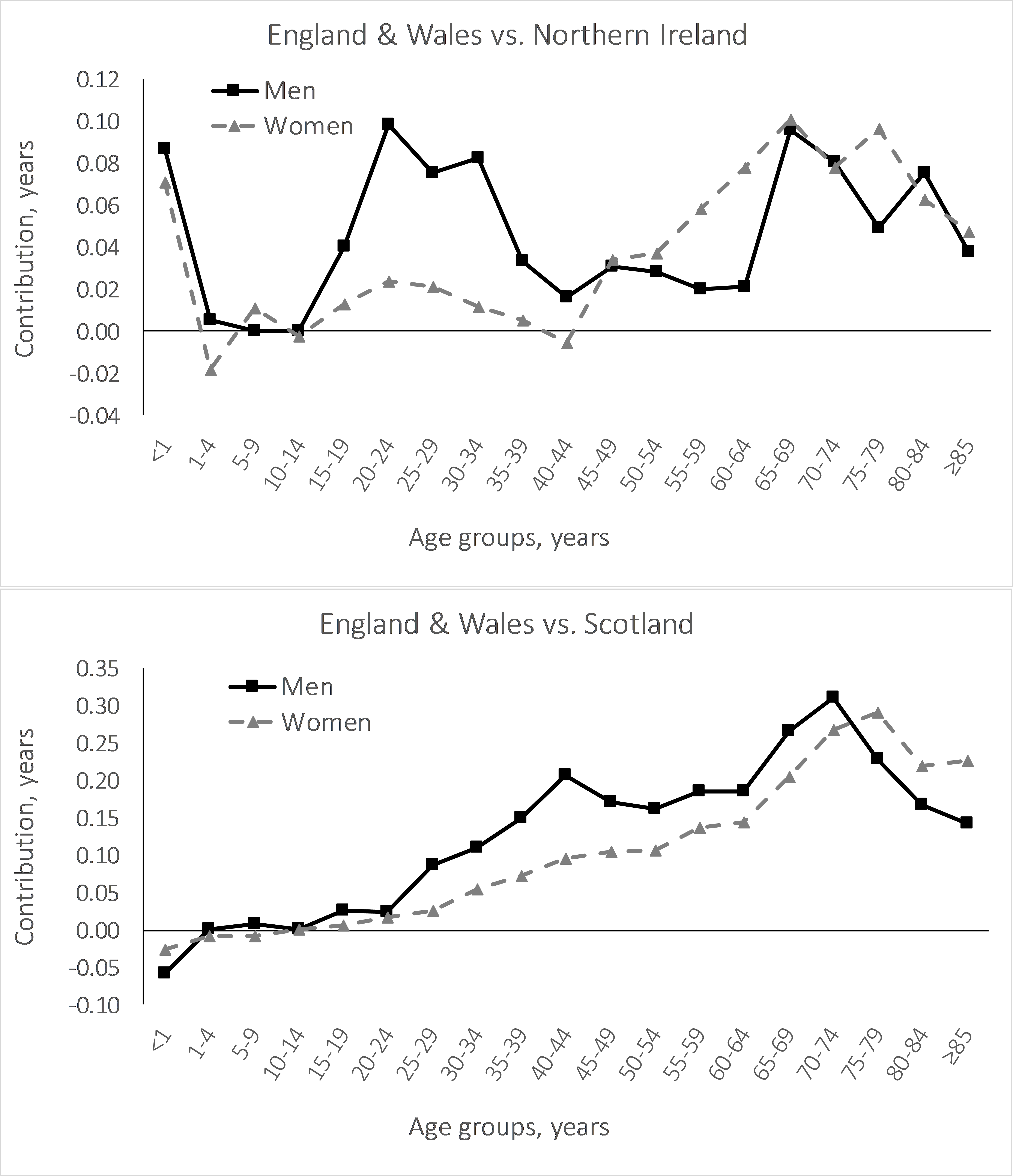

Supplement: Supplementary file 4 — Additional file 4. Age-specific contributions to the gap in life expectancy between England & Wales and the rest of the UK, by sex. Positive (negative) values indicate life expectancy advantage (disadvantage) in England & Wales. [file 12963_2021_271_MOESM4_ESM.tif]
